# The Effect of Core Stabilization Exercise on the Kinematics and Joint Coordination of the Lumbar Spine and Hip During Sit-to-Stand and Stand-to-Sit in Patients With Chronic Nonspecific Low Back Pain (COSCIOUS): Study Protocol for a Randomized Double-Blind Controlled Trial

**DOI:** 10.2196/resprot.7378

**Published:** 2017-06-01

**Authors:** Mohammad Reza Pourahmadi, Ismail Ebrahimi Takamjani, Shapour Jaberzadeh, Javad Sarrafzadeh, Mohammad Ali Sanjari, Holakoo Mohsenifar, Rasool Bagheri, Morteza Taghipour

**Affiliations:** ^1^ School of Rehabilitation Sciences Department of Physical Therapy Iran University of Medical Sciences Tehran Islamic Republic Of Iran; ^2^ School of Primary Health Care, Faculty of Medicine, Nursing and Health Sciences Monash University Peninsula Campus Melbourne Australia; ^3^ School of Rehabilitation Sciences Department of Rehabilitation Basic Sciences Iran University of Medical Sciences Tehran Islamic Republic Of Iran; ^4^ Department of Physical Therapy University of Social Welfare and Rehabilitation Sciences Tehran Islamic Republic Of Iran

**Keywords:** kinematics, lumbar spine, hip, chronic nonspecific low back pain, joint coordination, core stabilization exercise

## Abstract

**Background:**

Chronic nonspecific low back pain (CNLBP) is among the most prevalent health problems. Lumbar spine and hips kinematics and coordination can be affected in CNLBP. The effects of exercises on the kinematics and coordination of lumbar spine and hips during sit-to-stand (STS) and its reverse have not been evaluated.

**Objective:**

The aim of this study is to investigate the effect of core stabilization exercise on the kinematics and joint coordination of the lumbar spine and hip during STS and its reverse in CNLBP patients.

**Methods:**

COSCIOUS is a parallel randomized double-blind controlled trial. A total of 30 CNLBP patients and 15 asymptomatic participants will be included. The kinematics and joint coordination of the lumbar spine and hips will be evaluated during STS and its reverse using a motion capture system. The participants will be asked to sit in their usual posture on a stool. Reflective markers will be placed over the T12, S2, anterior and posterior superior iliac spines, greater trochanters, and lateral femoral epicondyles of both legs. The participants will be instructed to stand up at natural speed, remain in the erect posture for 3 seconds, and then sit down. Kinematic variables of the lumbar spine and hip will be computed. Afterward, the CNLBP participants will be allocated at random to receive one of 2 interventions: core stabilization or general exercise. Treatment sessions will be held 3 times per week for 16 sessions. After intervention, CNLBP participants will be assessed again.

**Results:**

Funding for the study was provided in 2016 by Iran University of Medical Sciences. The study is expected to last approximately 12 months, depending on recruitment. Findings on the study’s primary outcomes are expected to be finalized by December 2017. The results of the study will be published in a peer-reviewed journal.

**Conclusions:**

This investigation will evaluate the effects of core stabilization exercise on the kinematics and joint coordination of the lumbar spine and hip during STS and its reverse in patients with CNLBP. In addition, the effects of CNLBP on STS and its reverse will be investigated in COSCIOUS.

**Trial Registration:**

Iranian Registry of Clinical Trials IRCT2016080812953N2; http://en.search.irct.ir/view/32003?format=xml (Archived by WebCite at http://www.webcitation.org/6qjTWd4Az)

## Introduction

Low back pain (LBP) is defined as pain between the 12th rib and inferior buttock crease with or without leg pain [1,2]. According to the Global Burden of Disease Study, LBP is a major cause of years lived with disability in all continents and economies [3], and it is one of the main causes of absenteeism in industrialized societies [4]. Chronic low back pain (CLBP) is generally defined by symptoms that persist for a period of longer than 3 months (12 weeks) [5,6]. However, there is no precise definition of CLBP in the literature [7]. In some studies, CLBP is described as pain that lasts longer than 7 to 12 weeks, whereas the others define this condition as pain that lasts longer than expected with conventional treatment [7]. Due to no consensus regarding the definition of CLBP, there is a wide variation in the prevalence estimates reported in the literature [8]. However, Parthan et al [8] mentioned that the prevalence of CLBP ranges from 4% to 14%.

LBP is frequently associated with changes in the mobility of the lumbosacral region and hip [9-12]. Normal spinal mobility is required for optimal activities of daily living performance, and it has been shown that impairment of spinal mobility can result in various forms of functional disabilities [13], which may have serious adverse effects on quality of life [12]. Patients with LBP have been shown to have some limitations in both spinal and hip motion that compromise their function. In addition, Marras and Wongsam [14] indicated that the coordination between the lumbosacral region and hip is decreased during standing forward bending in patients with LBP. However, their simple descriptions of range of motion (ROM) and a simple forward bending task that do not adequately explore coordination between movements of the lumbosacral region and hips limit the generalizability of their findings to the functional tasks [12-14].

Sit-to-stand (STS) movement and its reverse, which are considered fundamental prerequisites for daily activities, are repeated many times throughout the day [12]. It has been shown that this movement could be performed about 60 times per day in workers [15]. In addition, STS movement is a skill [16], and the ability to perform this movement is a key factor in the maintenance of functional independence [17]. The manner in which STS movement is described depends somewhat on the aim of the study. For example, in the Roebroeck et al [18] study, STS movement is defined as traveling the body’s center of mass (COM) upward from a sitting position to a standing position without losing balance [18]. Vander Linden et al [19] defined STS movement as a transitional movement to the upright posture and requiring movement of the COM from a stable position to a less stable position over extended lower extremities. Schenkman et al [20] defined STS movement in more detail and divided it into 4 phases. Phase I (or *flexion-momentum phase*) begins with initiation of the movement and terminates just before the buttocks are lifted from the seat of the chair (lift-off). During this phase, the trunk and pelvis rotate anteriorly and the femurs, tibias, and feet remain stationary [20]. Phase II (or *momentum-transfer phase*) starts when the buttocks are lifted and terminates when maximal ankle dorsiflexion is achieved. During this phase, the COM moves anteriorly and upward [20]. Phase III (or *extension phase*) is begun just after maximum ankle dorsiflexion and completes when the hips first cease to extend, including leg and trunk extension. This phase is terminated when the hip extension angular velocity reaches 0° per second [20]. Phase IV (or *stabilization phase*) initiates after hip extension angular velocity reaches 0° per second and terminates when all motion associated with stabilization is completed [20]. Furthermore, Shum et al [12] divided STS movement into 2 phases: flexion and extension. The flexion phase begins with initiation of the movement and terminates when maximum flexion of the lumbar spine and hips is achieved (first 2 phases of Schenkman et al [20] classification). The extension phase starts just after phase I and continues until the end of the movement (last 2 phases of Schenkman et al [20] classification).

In 2005, Shum et al [12] evaluated the kinematics and joint coordination of the lumbar spine and hip during STS and its reverse in patients with acute LBP using an electromagnetic tracking device (3SPACE Fastrak system). The results of their study indicated that the mobility of the spine and hips and contribution of the lumbar spine relative to the hip were decreased in individuals with acute LBP. Furthermore, the lumbar spine–hip joint coordination was significantly altered in acute LBP subjects [12]. However, this study was conducted on acute LBP patients, and the data obtained from these participants is not representative of the population with chronic nonspecific low back pain (CNLBP). In addition, Shum et al [12] evaluated only the kinematics and joint coordination, and the effect of intervention on the kinematics has not been assessed. They recommended that further research is needed to evaluated the effect of intervention.

A core stabilization exercise program is described as facilitation of the deep musculature of the spine (primarily the transversus abdominis or multifidus) at low-level sustained isometric contraction, integrated into exercise and finally, progressing into functional tasks. The core stabilization exercise uses motor learning principles to facilitate coordination of the deep musculature of the spine [21-23]. It is usually delivered in 1:1 supervised treatment sessions and sometimes comprises palpation or ultrasound imaging or the use of pressure biofeedback units to provide feedback about the activation of trunk musculature [23].

To the best of authors’ knowledge, this study for the first time aims to investigate the effect of core stabilization exercise on the kinematics and joint coordination of the lumbar spine and hip during STS and its reverse in patients with CNLBP (COSCIOUS). The main objectives of COSCIOUS are as follows:

- To determine the kinematics and joint coordination of the lumbar spine and hip during STS and its reverse in patients with CNLBP and asymptomatic participants
- To determine the effect of core stabilization exercise on the kinematics and joint coordination of the lumbar spine and hip during STS and its reverse in patients with CNLBP

Macedo et al [24] mentioned that core stabilization exercise can optimize the control and coordination of the spine. However, there is not sufficient evidence to clearly support this claim that core stabilization exercise has superior effects on joint coordination compared to general exercise. Therefore, the secondary objective of this study is to determine the effect of general exercise on the kinematics and joint coordination during STS and its reverse in CNLBP. We hypothesized that there are significant differences between the results in patients with CNLBP and healthy participants during STS and stand-to-sit movements. In other word, ROM, maximum displacement, and angular velocity of the lumbar spine and hips would be decreased in CNLBP. Furthermore, we hypothesized that exercise therapy would have a significant positive impact on the kinematics and coordination in CNLBP patients. It is hoped that COSCIOUS would help clinicians and physical therapists evaluate the functional disabilities of patients with CNLBP, who are a big population in LBP patients. In addition, we hope that if this study exercise therapy protocol has a more positive effect on the kinematics and joint coordination, it can be used as a guideline for the treatment of functional disabilities (especially this important functional movement) in populations with CNLBP.

## Methods

### Study Design and Ethics

COSCIOUS is a parallel, randomized, double-blind controlled trial with 2 experimental groups and one asymptomatic (control) group. It is a part of the PhD thesis of the first author (MRP) and will be conducted in the biomechanics laboratory located at the School of Rehabilitation Sciences, Iran University of Medical Sciences (Tehran, Iran). The level of evidence of this investigation is level Ib. Ethical review and approval for COSCIOUS was obtained from the Research Ethics Committee at Iran University of Medical Sciences (Ethical Approval Number: IR.IUMS.REC 1395.9211342207) on July 27, 2016. The trial is registered at the Iranian Registry of Clinical Trials [IRCT2016080812953N2].

### Participants

A total of 30 patients with CNLBP and 15 demographically matched asymptomatic (healthy) participants will be recruited for COSCIOUS. The sample size of CNLBP patients is determined based on previous studies that have evaluated the effect of lumbar extension exercise on the kinematics of the lumbar spine and hips during a common daily activity [25,26]. Steele et al [26] calculated the sample size for their study and reported that a minimum of 7 participants would be required in each group to achieve 80% power at an alpha level of *P* ≤.05 [26]. Therefore, 45 participants will be divided into 3 groups: 2 experimental groups (15 patients with CNLBP in each group) and one healthy control group (15 asymptomatic participants). Considering some attrition, the sample size was increased to 15 participants in each group. The CNLBP patients will be identified and recruited by posters, emails, and word of mouth from the university and the surrounding locality. Direct referral also will be provided from 2 orthopedic surgeons and 2 neurologists in Tehran. Inclusion criteria of COSCIOUS are as follows:

- CNLBP (LBP persisting for more than 3 months in the absence of an underlying pathology)
- Aged between 18 and 40 years
- Pain between 3 and 6 at rest on a 0- to 10-point pain visual analog scale (VAS), where 0 represents no pain and 10 is the worse pain imaginable
- Ability to perform STS movement and its reverse without an aid
- No contraindication for exercise
- No obvious deformity of the spine, pelvis, and lower extremities
- No autoimmune diseases (eg, rheumatoid arthritis) [12]
- No pregnancy

In this study, we will try to balance some of most important prognostic indicators of LBP such as educational status, smoking, working status, age, gender, and pain intensity in 2 treatment groups [27,28]. This strategy will help minimize the effects of confounding variables. Participants who do not complete the treatment sessions (absence for 3 consecutive or a total of 5 sessions) will be excluded from the study. Before participation in this study, procedure of this investigation will be explained both verbally and in writing, and written informed consent will be obtained from all participants. In addition, the participants will be assured that their personal information will be kept confidential (confidentiality principle). Participants will be identified only by initials and participant number on the research notes. All study results and completed questionnaires will be anonymized. All trial documents will be stored securely and not accessible to people outside of the research team. Participants will be withdrawn from this investigation if there are any concerns regarding informed consent.

### Randomization

Each CNLBP participant will be randomized to a general exercise group or a core stabilization exercise group with a ratio of 1:1. Randomization will be performed using a block-balanced randomization technique with 4 character blocks containing letters A and B. After randomizing, the randomization schedule will be transferred into written instructions and will be placed in sequentially numbered, opaque, sealed envelopes. The CNLBP participant will be blinded to which intervention group they are in until the end of the study. The procedure will be performed by an investigator who will not be involved with participant assessment, allocation, or treatment. The randomization schedule is known only to a physical therapist who treats the patients. The male physical therapist (MRP) assessing participants before and after the treatment will also be blinded to group allocation, and the patients will be asked not to disclose this to the physical therapist.

### Examiners

Participants will be evaluated by a male physical therapist (MRP) who is a doctoral candidate with more than 6 years of clinical experience. In addition, CNLBP patients’ treatment will be performed by a female physical therapist who is a master’s degree student with more than 10 years of clinical experience in orthopedic physical therapy and certified in manual therapy.

### Equipment

Pain will be measured by the use of a 100 mm point VAS, and functional disability will be measured using the Persian translated version of the Ronald-Morris Disability Questionnaire (RMDQ). The RMDQ is a self–administered disability measure that was first published in 1983 [29]. It provides a tool for measuring the level of functional disability experienced by a patient suffering from LBP. The RMDQ includes 24 statements relating to the individual’s perceptions of their LBP and associated disability divided into 6 domains: physical ability and activity (15), sleep and rest (3), psychosocial (2), household management (2), eating (1), and pain frequency (1) [29]. The RMDQ is designed to take about 5 minutes to complete, without any assistance from the administrator. Validity and reliability of the Persian translated version of the RMDQ has been evaluated by Mousavi et al [30], who reported high correlation between the results of this questionnaire and the physical functioning scales of the Short Form Health Survey (*r*=–0.62) and excellent reliability (intraclass correlation coefficient =0.86). In addition, Mousavi et al [30] concluded that the Persian translated version of the RMDQ can measure functional status in Persian-speaking patients with LBP. The RMDQ has been selected for measuring functional disability because Parthan et al [8] stated that the RMDQ is the most responsive instrument and is recommended for clinical trials in CLBP.

STS and its reverse kinematic variables will be captured at a sample frequency of 100 Hz using good resolution (1.3 megapixel) cameras and a 3-dimensional motion capture system (Qualisys AB). Kinematic data will be analyzed using Qualisys track manager (QTM) software (Qualisys AB), MatLab version R2015b (MathWorks Inc), and Excel version 2016 (Microsoft Corp).

### Motion Capture System

A 3-dimentional approach will be used for data collection. However, only kinematic data in the sagittal plane will be used for analysis. According to Shum et al [12], motions out of the sagittal plane have insignificant amplitudes and cannot be used for analysis. A total of 6 Oqus 300 cameras (Qualisys AB) will be set up and angled in a manner to decrease hidden spots that might obscure data collection. In addition, the QTM software will be used to synchronize the 6 Oqus 300 cameras that record marker motion. The cameras identify infrared reflective markers which are attached to the participants and output 3-dimensional coordinates for each marker. Prior to testing, the motion system will be calibrated by recording an L-frame and dynamic movement of a T-wand for 60 seconds. After data collection, the markers will be labeled according to the anatomical landmark to which each marker is attached.

### Marker Setup

A total of 14 reflective markers will be placed on anatomical landmarks. Lumbar spine segment is modeled by placing 2 25.4 mm spherical markers on the T12 and S2 spinous processes [31]. The intercrestal [32] or intercristal line [33]—the line joining the superior aspect of the iliac crests posteriorly—will be used to find the L3 spinous process or L3-L4 spinal level [34,35]. Once the L3 spinous process or L3-L4 spinal level is identified, the physical therapist will palpate the spinous processes in the midline and trace them from inferior to superior to find the T12 spinous process. The posterior superior iliac spines (PSISs) line—the line joining the right and left PSISs—will also be used to find the S2 spinous process [34]. Furthermore, 4 14.0 mm spherical markers will be placed on the anterior superior iliac spines (ASISs) and PSISs of both sides to the define pelvis segment. In order to define femur segments, 8 14.0 mm spherical markers will be placed on the right and left greater trochanters, lateral femoral epicondyles, calcaneuses, and base of the 5th metatarsals. Markers will be placed by the same investigator (MRP) for all trials. Marker trajectories will be used to calculate the position and orientation of the anatomical frames of the 4 segments (pelvis, lumbar, and 2 hips). Sagittal lumbar angle is defined as the angle between the lumbar and pelvis segments. In addition, sagittal right hip (RH)/left hip (LH) angles are defined as the angle between the right and left femur and pelvis segments. The joint coordinate system [36] will be used to calculate sagittal joint angles.

### Procedure

#### Initial Assessment

**Figure 1:**
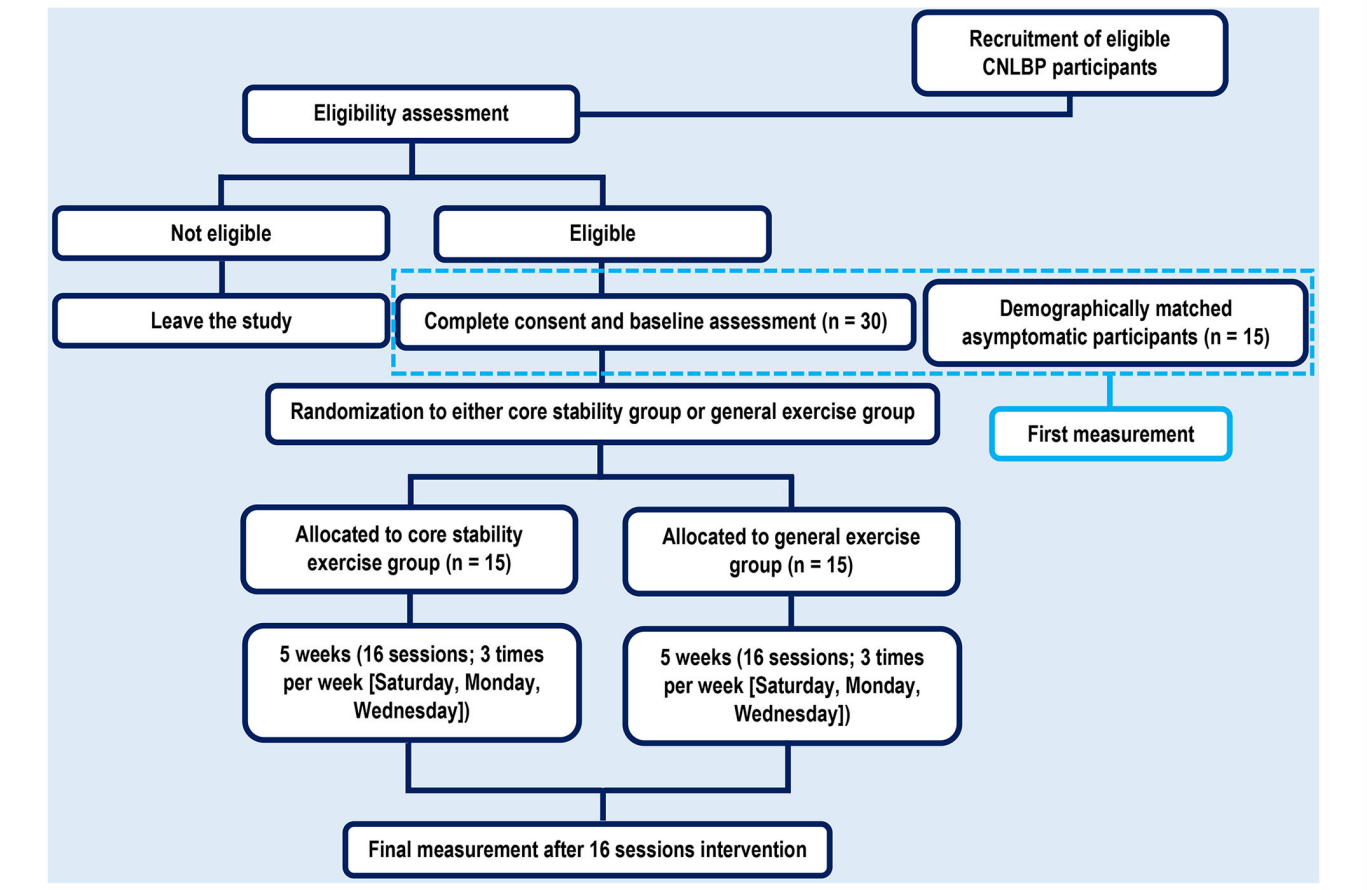
CONSORT diagram illustrating flow of participants through the COSCIOUS.

After the recruitment process is complete and agreement to participate is obtained, participants will be brought into the biomechanics laboratory of the School of Rehabilitation Sciences, Iran University of Medical Sciences (Tehran, Iran). To ensure the activity is as natural as possible, few constraints will be placed on the procedure of STS movement and its reverse. Participants will not be allowed to use their hands to push up, and the feet should stay on the floor [12]. In order to standardize head posture and prevent the negative impact of the thoracic and cervical spines on lumbar spine kinematics, a visual target (white A4 paper with a black circle drawn upon it) will be adjusted to eye level in front of participants [31]. Data collection will begin with the capture of a reference standing posture. Then, participants will be asked to sit in their usual posture on an adjustable stool with neither armrest nor backrest. The stool provides support from the ischial tuberosities to the middle of the thighs. Foot placement will not be restricted and participants will be requested to relax their arms, hanging them next to their thighs. The stool height is 110% of knee-floor length, which is defined as the distance between the floor and the apex of the fibular head [12]. Participants will be instructed to stand up at their self-selected normal speed following a loud voice command (“Start”) and remain a comfortable and erect posture for 3 seconds. They will then be asked to sit down on the chair at their own comfortable speed and maintain this position for 3 seconds. Each subject will repeat the movements 3 times, and the mean data will be used for statistical analysis. Asymptomatic participants will serve as a control group in the first assessment in order to detect differences in kinematic parameters during STS and its reverse between the CNLBP patients and asymptomatic participants. Hence, they will not participate in the intervention phase and the second assessment (Figure 1).

Following the assessment, CNLBP participants will be randomly divided into 2 groups (core stabilization exercise and general exercise) for treatment. First, all CNLBP participants will perform warm-up exercises that consist of 8 stretching exercises (hamstrings, quadriceps, latissimus dorsi, and iliotibial band of both sides; 1 set of 5 repetitions with a 10-second hold per repetition) and stationary bicycling for a period of 10 minutes [37,38]. Based on the previous recommendations, a staged approach will be followed for both groups [38] (see Multimedia Appendix 1). This approach includes 8 exercise levels with progressively increasing difficulty [38]. In the first session, the exercises will be explained and demonstrated to CNLBP participants. We will try to use the Template for Intervention Description and Replication checklist [39] as a guideline to improve the completeness of reporting of our intervention procedure.

#### Core Stabilization Exercise

Core stabilization or motor control exercise is a common type of therapeutic exercises prescribed for LBP patients. Core stabilization exercise is designed to re-educate the coactivation pattern of abdominals, paraspinals, gluteals, pelvic floor muscles, and diaphragm [23,40]. The biological rationale for core stabilization exercise is primarily based on the idea that the stability and control of the spine are altered in patients with LBP [41]. A core stabilization exercise program begins with recognition of the natural position of the spine (midrange between lumbar flexion and extension ROM), considered to be the position of balance and power for improving performance in various sports [42]. Initial low-level sustained isometric contraction of trunk stabilizing muscles and their progressive integration into functional tasks is the requirement of core stabilization exercise [21]. To ensure correct activation of the transversus abdominis muscle, it will be emphasized to CNLBP participants that the lower part of the anterior abdominal wall below the umbilical level needs to be “drawn in” during contraction of this muscle. Furthermore, bulging action of the multifidus muscle needs to be felt under the physical therapist’s fingers when they are placed on either side of the spinous processes of the L4 and L5 lumbar vertebral levels, directly over the belly of the muscle [37]. Participants will be made aware of and will be told to avoid incorrect muscle activation (substitution strategies).

#### General Exercise

General exercise is an umbrella term that can involve strengthening exercise for all the main muscle groups with or without the addition of weights [43]. In addition, this umbrella term can consist of exercises improving coordination, stretching, and aerobic fitness training [43]. In our study, exercises activating the extensor (paraspinals) and flexor (abdominals) muscle groups will be performed in a lying position initiating with simple movements and progressing to more difficult exercises (eg, on a gym ball).

The same frequency (*3 times per week*; Saturday, Monday, Wednesday) and duration (6 weeks; 16 sessions) will be provided for both groups [37]. All treatment sessions will be held in the morning (8 AM to 12 PM). In each session, participants will be instructed to perform their exercises as many times as they can with rest periods in the same session. However, the holding time and number of contractions will be progressively increased up to 10 contraction repetitions × 10 second duration each [38]. Finally, exercise sets will be increased from 3 to 5 sets. The first session will last for 30 to 45 minutes, and the time of treatment session will be gradually increased in a dose-dependent manner. Based on Koumantakis et al [38] study, CNLBP participants in the core stabilization exercise group will be asked to activate their muscles at about 30% of their maximum activation level during the performance of stabilization exercises, and CNLBP participants in the general exercise group will be asked to activate their muscles at about 60% to 70% during the performance of general exercises. However, in the core stabilization exercise group, heavier load functional tasks, with exercises similar to those conducted by CNLBP participants in the general exercise group, will be progressively introduced in the last 6 sessions of the program [37]. All exercises will be performed under supervision of an experienced physical therapist. Moreover, to control confounding variables and create a standard and homogeneous condition for all CNLBP participants, they will be instructed not to perform exercises at home between the treatment sessions. Treating physical therapist will explain the positive effects of exercise on health in each session to improve adherence of CNLBP participants to intervention protocol. Any deviations from the exercise protocol, such as the receipt of any additional interventions or therapy for LBP, will be recorded and the decision will be made on whether the participant should be excluded from the study.

After 16 treatment sessions, all CNLBP participants will be evaluated again in the same manner as the initial assessment. Pain intensity and functional disability will be measured using the VAS and the Persian translated version of the RMDQ. Figure 1 illustrates a Consolidated Standards of Reporting Trials (CONSORT) diagram of this study.

### Management of Adverse Events

CNLBP participants will be encouraged to contact their treating physical therapist between appointments if they have any concerns about their exercise program or if they experience an increase in pain. These concerns will be addressed by their treating physical therapist and details of the issue and outcome recorded [44]. Any adverse events will be recorded and reported to the ethics committee at Iran University of Medical Sciences.

### Main Outcome Measures

#### Kinematic Analysis

The maximum flexion and extension ROM, the timing of their occurrence, and angular velocities of the lumbar spine and right and left hips will be computed in asymptomatic and CNLBP participants during flexion and extension phases [12] of STS and its reverse. The ratios of the total movements of the lumbar spine to those of the right hip and to the left hip will be determined during STS movement and its reverse. These ratios indicate the relative contributions of the joint pairs throughout the ROM. These kinematic parameters will be assessed again in both intervention groups after intervention. Each STS and its reverse will be screened to determine visually the beginning and the end of the movement. A single investigator will follow the same rule for every participant; start of T12 anterior displacement and stop of S2 displacement. Sagittal lumbar and hip angles will be calculated following filtering of the raw data using a robust low-pass Butterworth filter (6 Hz) [45].

#### Relative Phase Angle Analysis

Relative phase angle (RPA) is considered a technique to quantify coordination patterns and variability in the dynamical systems theory approach [46]. The RPA can represent both temporal and spatial information continuously throughout the STS movement and its reverse [46]. The RPA will be used to measure interjoint coordination between the lumbar spine and dominant hip. Phase angle is defined as the inverse tangent of angular velocity/angular displacement and will be calculated for each data point through the entire cycle (phase angle *φ* tan^–1^ [angular velocity/angular displacement]). The RPA between 2 body segments, which represents the joint coordination, will be calculated continuously from the differences between the phase angles of 2 joints (dominant hip–lumbar spine) [12,46]. The dominant leg will be determined by asking the participants with which leg they prefer to kick a ball [47]. If the RPA is negative, the hip is lagging the lumbar spine, and if the phase difference is positive, the hip movement is leading the lumbar spine [12]. Maximum and minimum relative phase differences and their timing will be computed before and after intervention in CNLBP participants. Furthermore, the relative phase relationship between the lumbar spine and hips (relative phase difference; x-axis) versus each percent of movement (y-axis) for STS and its reverse will be displayed in plots before and after intervention.

### Statistical Analysis

Statistical analyses will be performed on a personal laptop using SPSS for Windows release version 21.0 (IBM Corp) and STATA version 13 (StataCorp LLC). The comparability of asymptomatic participants and CNLBP patients on disability level, pain intensity, and kinematic variables at baseline will be analyzed using the independent *t* tests for parametric and Wilcoxon test for nonparametric distribution. In addition, a mixed-model 2-way analysis of variance (ANOVA) for each dependent variable (disability level, pain intensity, maximum joints ROM, joint angular velocities, lumbar/hip motion ratios, and maximum and minimum relative phase differences), with the 2 factors being group (core stabilization/general exercise) and time (preintervention/postintervention). After intervention, comparison between core stabilization group, general exercise group, and asymptomatic participants will be made using the ANOVA test (*Scheffe* post hoc tests). In addition, an intention-to-treat analysis will be conducted if any protocol deviation is observed at the end of the study due to attrition or loss to follow-up. Perceived pain and functional disability will be compared with consensus standards for minimal clinically important change (MCIC) [48]. Ostelo et al [48] considered the MCIC for VAS as 15 mm and for RMDQ as 5 points for patients with LBP. Furthermore, effects sizes will be measured using the Cohen *d* [49]. Statistical significance level is set at *P* ≤.05.

## Results

COSCIOUS should be completed in December 2017. The results of this study will be submitted to a peer-reviewed MEDLINE-indexed journal for publication and will be presented at national and international academic and clinical conferences.

## Discussion

### Overview

To the best of the authors’ knowledge, this is the first time an investigation will evaluate the effects of core stabilization exercise on the kinematics and joint coordination of the lumbar spine and hip during STS and its reverse in patients with CNLBP. STS movement and its reverse are important functions required for conducting activities of daily living. Previous study has shown that these skills would be impaired in acute LBP [12]. However, the effects of CNLBP on flexion and extension phases of STS and its reverse is not well known, and COSCIOUS aims to investigate this issue.

Physical therapy programs play a key role in multimodal treatment approaches for CNLBP [50]. Therapeutic exercise is one of the few clearly effective physical therapy treatments to relieve pain for adults with CNLBP [50,51]. The European guidelines for the management of CNLBP suggest supervised exercise therapy as a first-line treatment [52]. In this investigation, the effect of the 2 most common types of therapeutic exercises prescribed for patients with LBP will be evaluated on the kinematics and joint coordination of functionally important activities. To the best of authors’ knowledge, no published study has assessed the effect of exercise therapy on STS movement and its reverse. The main study question is whether core stabilization exercise programs are effective in improving kinematic variables during STS and its reverse in CNLBP. In addition, a comparison will be made between the results of the 2 therapeutic exercises on STS and its reverse. The results of COSCIOUS will provide some insight into the effects of CNLBP on lumbar spine–hip coordination of activities of daily living.

### Limitations

The main limitation of this study is that young and middle-aged CNLBP participants will be included, thereby, the generalizability is limited. In addition, some study participants may be lost to follow-up.

### Conclusion

COSCIOUS will evaluate the effects of core stabilization exercise on the kinematics and joint coordination of the lumbar spine and hip during STS and its reverse in patients with CNLBP. In addition, the effects of CNLBP on the STS and its reverse will be investigated.

## Appendix

Multimedia Appendix 1 Core stabilization and general exercise programs.

## Abbreviations

ANOVA: analysis of variance
ASIS: anterior superior iliac spine
CLBP: chronic low back pain
CNLBP: chronic nonspecific low back pain
COM: center of mass
CONSORT: Consolidated Standards of Reporting Trials
COSCIOUS: core stabilization exercise on the kinematics and joint coordination of the lumbar spine and hip during sit-to-stand and its reverse in patients with chronic nonspecific low back pain
LBP: low back pain
LH: left hip
MCIC: minimal clinically important change
PSIS: posterior superior iliac spine
QTM: Qualisys track manager
RH: right hip
RMDQ: Ronald-Morris Disability Questionnaire
ROM: range of motion
RPA: relative phase angle
STS: sit-to-stand
VAS: visual analog scale
