# Supplementary material for: The Effect of Core Stabilization Exercise on the Kinematics and Joint Coordination of the Lumbar Spine and Hip During Sit-to-Stand and Stand-to-Sit in Patients With Chronic Nonspecific Low Back Pain (COSCIOUS): Study Protocol for a Randomized Double-Blind Controlled Trial
Source: JMIR Res Protoc. 2017 Jun 1;6(6):e109. doi: 10.2196/resprot.7378 (PMC5472842; doi:10.2196/resprot.7378)
Supplement: Multimedia Appendix 1 [file resprot_v6i6e109_app1.pdf]

Appendix:

Stage 1		
Session	**Core Stability Group**<br>***Isolated lumbar stabilizing muscle training***	**General Exercise Group**<br>***Classic abdominal and back extensor training***
1-2	Development of the perception of the isolated isometric specific contraction of the stabilizing muscles<br>Transversus abdominis muscle<br>from: 4-point kneeling and lying positions, trying to hollow the lower abdomen<br>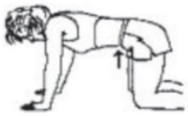<br>Multifidus muscle from: stepping activity while standing and raising contralateral arm, trying to feel the contraction of the opposite-side multifidus muscle or from sitting position with therapist's hands over the muscle<br>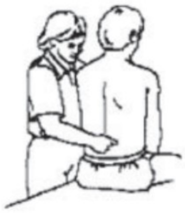	Upper and oblique abdominals from lying position: with Knees straight (hands filling space between low back and exercise mat) and knees bent<br>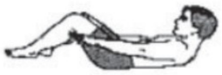<br>Back extensors: lifting trunk to neutral from prone position with pillow under stomach and arms by the side<br>Coordination: pelvic tilting from lying, sitting, and standing positions<br>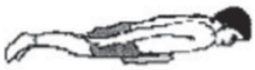
3-4	Precise repetition of the isolated isometric-specific co-contraction of the stabilizing muscles, increasing their contraction time<br><br>Transversus abdominis and multifidus muscles together from: sitting and standing positions<br>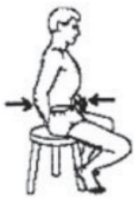	Upper and oblique abdominals from lying position: with knees straight, knees bent<br><br>Back extensors: lifting trunk to neutral from prone position with pillow under stomach and arms by the side Exercises performed as illustrated for sessions 1 and 2

Stage 2		
Session	**Core Stability Group**<br>***Integration of lumbar stabilizing muscle activity into light dynamic functional tasks***	**General Exercise Group**<br>***Classic abdominal and back extensor training***
5-6	Control of neutral lumbopelvic postures<br>Isolated movement of adjacent body areas, maintaining lumbar spine stability (ie, moving only hip or thoracic spine)<br>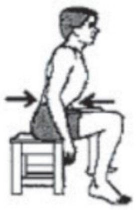	Abdominals from lying position: heel slides, lower abdominal crunches<br><br>Back extensors: bridging, lifting trunk to neutral from prone position and arms in elevation<br>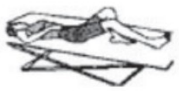<br>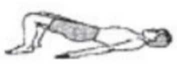
7-8	Control of neutral lumbopelvic postures and aggravating postures<br><br>Stabilizing muscle isometric co-contractions with addition of external load to lumbar spine<br><br>Hip horizontal abduction, heel slides, leg slides from crook-lying position<br><br>Aggravating postures<br>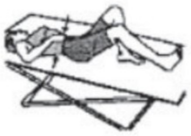	Abdominals from lying: heel slides, leg slides, lower abdominal crunches<br><br>Back extensors: bridging, lifting trunk to neutral(prone position with arms elevated), single-leg extensions from prone and 4-point kneeling positions<br>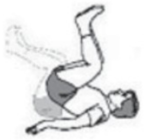<br>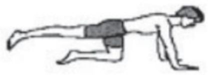
9-10	Lumbopelvic control during movements and aggravating movements<br><br>Sitting on unstable base of support (hip extension movement only, lumbar spine only, thoracic only), 3-plane movement, co-contractions during normal-speed walking and other activities<br>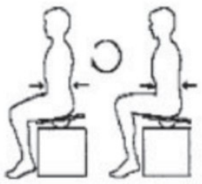	Abdominals from lying position: straight leg lifts toward ceiling, cycling exercises, leg slides, lower abdominal crunches<br><br>Obliques: hip lift from side-lying position<br><br>Back extensors: as in sessions 7-8<br>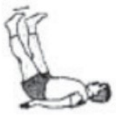<br>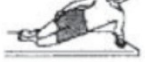

	Stage 3	
Session	Core Stability Group<br>Integration of lumbar stabilizing muscle activity into heavy-load dynamic functional tasks	General Exercise Group<br>Classic abdominal and back extensor training
11-12	Isometric co-contractions with addition of heavier external loads to lumbar spine<br><br>Bridging exercise, co-contractions during leg cycling from supine position, single-leg extensions from 4-point kneeling position<br>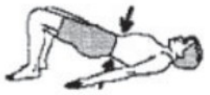<br>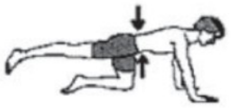	Abdominals from lying position: full abdominal crunches, straight leg lifts toward ceiling, cycling exercises, leg slides<br><br>Obliques: hip lift from side-lying position<br><br>Back extensors: alternate arm/leg extensions from 4-point kneeling and lying positions, single-leg bridging<br><br>Swiss ball coordination exercises: alternate arm/leg lifts sitting on ball<br>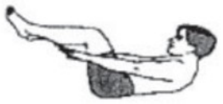<br>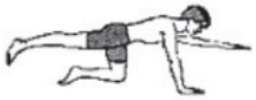
13-14	Increasing complexity and load of exercises maintaining lumbar spine stability<br><br>Single-leg bridging exercise, bridging exercise with an unstable base of support<br><br>Alternate arms/leg extensions from 4-point kneeling and lying positions and arm/leg lifts sitting on Swiss ball<br><br>Functional co-contractions during walking (increasing speed) and other activities	Abdominals from lying position: same leg and arm lifting-lowering, full abdominal crunches, straight leg lifts toward ceiling, cycling exercises, leg slides<br><br>Obliques: advanced hip lift from side-lying position<br><br>Back extensors: as in sessions 11-12<br><br>Swiss ball coordination exercises: abdominal curls on ball from prone position, pulling legs toward chest

13-14	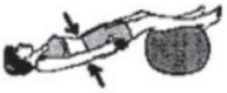 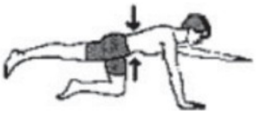	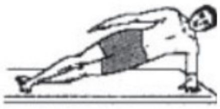 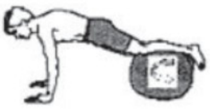
15-16	*Coordination exercises*<br><br>Single-leg bridging exercise with an unstable base of support, bridging exercise with rotator self-resistance, simultaneous arm and leg movements from supine position maintaining lumbar spine stability, functional co-contractions during walking (changing speeds) and other activities<br><br>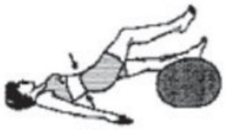	Abdominals from lying position: same leg and arm lifting-lowering, cycling exercises<br><br>Obliques: full oblique abdominal crunches,<br>lift from side-lying position<br><br>Back extensors: as in sessions 11-12<br><br>Swiss ball co-ordination exercises: oblique abdominal curls on ball from prone position, single-leg bridging<br><br>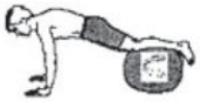 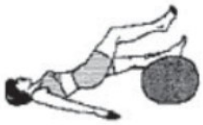

This protocol has been adopted from Shamsi et al. [37].
